# Tumor volume in subcutaneous mouse xenografts measured by microCT is more accurate and reproducible than determined by ^18^F-FDG-microPET or external caliper

**DOI:** 10.1186/1471-2342-8-16

**Published:** 2008-10-16

**Authors:** Mette Munk Jensen, Jesper Tranekjær Jørgensen, Tina Binderup, Andreas Kjær

**Affiliations:** 1 Cluster for Molecular Imaging, University of Copenhagen, Copenhagen, Denmark; 2 Rigshospitalet, Dept. of Clinical Physiology, Nuclear Medicine & PET, Copenhagen, Denmark

## Abstract

**Background:**

In animal studies tumor size is used to assess responses to anticancer therapy. Current standard for volumetric measurement of xenografted tumors is by external caliper, a method often affected by error. The aim of the present study was to evaluate if microCT gives more accurate and reproducible measures of tumor size in mice compared with caliper measurements. Furthermore, we evaluated the accuracy of tumor volume determined from ^18^F-fluorodeoxyglucose (^18^F-FDG) PET.

**Methods:**

Subcutaneously implanted human breast adenocarcinoma cells in NMRI nude mice served as tumor model. Tumor volume (n = 20) was determined *in vivo *by external caliper, microCT and ^18^F-FDG-PET and subsequently reference volume was determined *ex vivo*. Intra-observer reproducibility of the microCT and caliper methods were determined by acquiring 10 repeated volume measurements. Volumes of a group of tumors (n = 10) were determined independently by two observers to assess inter-observer variation.

**Results:**

Tumor volume measured by microCT, PET and caliper all correlated with reference volume. No significant bias of microCT measurements compared with the reference was found, whereas both PET and caliper had systematic bias compared to reference volume. Coefficients of variation for intra-observer variation were 7% and 14% for microCT and caliper measurements, respectively. Regression coefficients between observers were 0.97 for microCT and 0.91 for caliper measurements.

**Conclusion:**

MicroCT was more accurate than both caliper and ^18^F-FDG-PET for *in vivo *volumetric measurements of subcutaneous tumors in mice.^18^F-FDG-PET was considered unsuitable for determination of tumor size. External caliper were inaccurate and encumbered with a significant and size dependent bias. MicroCT was also the most reproducible of the methods.

## Background

Measurement of tumor size is important in preclinical animal studies when assessing responses to cancer treatment. In longitudinal studies, sequential measurements of tumor volume with a non-invasive method are essential. Current standard technique for volume determination of subcutaneously xenografted tumors *in vivo *is by external caliper where tumor volume is calculated by use of the modified ellipsoid formula 1/2(Length × Width^2^)[1,2]. However, measurements using caliper are often affected by errors due to *e.g. *variability in tumor shape, skin thickness and subcutaneous fat layer thickness. Furthermore, observer subjectivity and differences in the compressibility of the tumor can easily lead to variation in measurements. Clinically, computed tomography (CT) and positron emission tomography (PET) are widely used to monitor response to treatment [3]. Preclinical imaging with microCT and microPET has in recent years become more widespread [4-8].

The aim of the present study was therefore to evaluate if microCT gives more accurate and reproducible measures of tumor volume in *in vivo *studies of subcutaneous xenografted tumors compared with standard caliper measurements. Furthermore, we evaluated the accuracy of tumor volume determined from ^18^F-fluorodeoxyglucose (^18^F-FDG) PET. To do so, we compared the microCT, PET and caliper methods for tumor volume determination, with ex vivo measurements as reference, and quantified inter- and intra-observer variation for the microCT and caliper methods.

## Methods

### Tumor Model

Six weeks old female NMRI (Naval Medical Research Institute) nude mice were acquired from Taconic Europe (Lille Skensved, Denmark) and allowed to acclimate one week in the animal facility before any intervention was initiated. All experimental procedures were conducted with the guidelines set forth by the Danish Ministry of Justice. Estrogen pellets, 0.72 mg 17-β-Estradiol, 60-day release (Innovative Research of America, Sarasota, FL, USA), were implanted s.c. during anesthesia with 1:1 v/v mixture of Hypnorm^® ^(Janssen Pharmaceutica, Beerse, Belgium) and Dormicum^® ^(Roche, Basel, Switzerland). One week after implantation of pellets, MCF-7 (human breast adenocarcinoma) tumor cells (10^7 ^cells in 100 μL medium mixed with 100 μL Matrixgel™ Basement Membrane Matrix (BD Biosciences, San Jose, CA, USA)) were injected subcutaneous into the left and right flank respectively. Cells were cultured in Dulbecco's Modified Eagle Medium (DMEM) medium supplemented with 10% fetal calf serum and 1% penicillin-streptomycin in 5% CO_2 _at 37°C.

### Volume Determination

Three weeks after implantation of tumor cells (tumor size 20 – 250 mm^3^) volumes of 20 tumors were determined *in vivo *by external caliper, microCT and ^18^F-FDG-PET. Subsequently, tumors were excised and reference tumor volume was calculated from weight and density (1.05 g/mL).

In order to assess the intra- and inter-observer variation on the microCT and caliper volume measurements two additional experiments were carried out. To determine the intra-observer reproducibility, volume of two tumors was determined by acquiring 10 microCT scans and 10 caliper determinations of each tumor. In addition, volumes of a group of 10 tumors were determined independently by two different observers to assess inter-observer variation. Each of the 10 mice had one microCT scan performed and region of interests (ROIs) were subsequently drawn covering the tumors independently by each of the two observers.

### Measurement by Caliper

In order to determine tumor volume by external caliper, the greatest longitudinal diameter (length) and the greatest transverse diameter (width) were determined. Tumor volume based on caliper measurements were calculated by the modified ellipsoidal formula [1,2]

*Tumor volume *= 1/2(*length *× *width*^2^)

### ^18^F-FDG microPET imaging and microCT imaging

Mice were injected i.p or i.v with 8.7 ± 1.7 (mean ± SD) MBq of ^18^F-FDG. ^18^F-FDG was produced at our own facilities (Rigshospitalet, Copenhagen, Denmark). One hour after ^18^F-FDG injection mice were anaesthetized with 3% sevofluran (Abbott Scandinavia AB, Solna, Sweden) mixed with 35% O_2 _in N_2 _and fixed on a bed. A 20 min PET scan was acquired using a MicroPET Focus 120 (Siemens Medical Solutions, Malvern, PA, USA). After data acquisition, PET data were arranged into sinograms and subsequently reconstructed with the Ordered Subset Expectation Maximization 2D (OSEM2D) reconstruction algorithm. The pixel size was 0.866 × 0.866 × 0.796 mm and in the center field of view the resolution was 1.4 mm full-width-at-half-maximum.

Following the microPET scan, a microCT scan was acquired with a MicroCAT^® ^II system (Siemens Medical solutions). A 7 minute and 10 seconds CT scan was performed with parameter settings: 360 rotation steps, tube voltage 60 kV, tube current 500 μA, binning 4 and exposure time 310 ms. The pixel size was 0.081 × 0.081 × 0.081 mm.

PET and microCT images were separately analyzed with the Inveon software (Siemens). ROIs were drawn manually by qualitative assessment covering the entire tumor and tumor volume was generated by summation of voxels within the tomographic planes.

### Statistical analysis

Analysis of tumor volumes obtained by the three different methods was performed by linear regression for each method against reference tumor volume. Agreement between the PET, microCT and caliper methods against reference tumor volume was further analyzed by means of Bland-Altman plots where the central line (mean) indicates the bias and the outer lines (± 2SD) indicate the limits of agreements (LoA) [9]. The 95% confidence interval (CI) on bias was calculated, and bias was considered significant if 0 was not included in the CI.

In order to assess the intra-observer variation, coefficient of variation (CV) for the 10 repeated microCT and caliper measurements were calculated. Tumor volumes measured by two experienced observers were evaluated my means of linear regression, correlation coefficients and Bland-Altman plots in order to determine the inter-observer variation.

## Results

### Tumor volume determined by microCT, ^18^F-FDG-PET and external caliper

**Figure 1:**
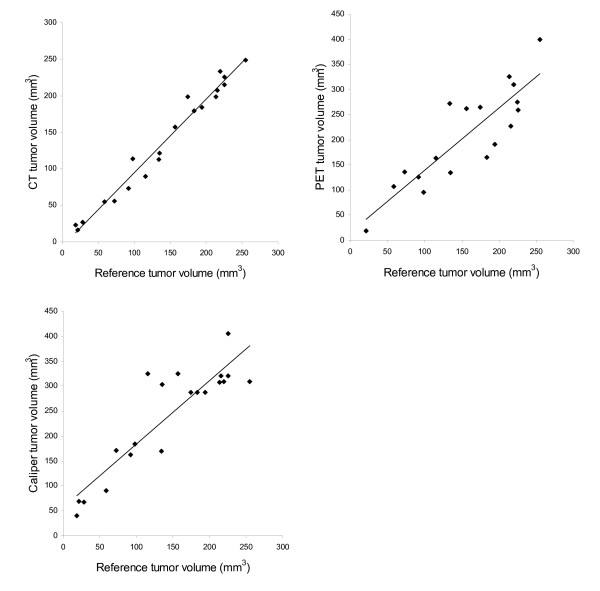
**Linear regression for microCT, ^18^F-FDG-PET and caliper determined tumor volume against reference tumor volume**. Best lines were: y = 1.01x - 6.1 (R^2 ^= 0.97) for microCT versus reference volume, y = 1.24x + 15.4 (R^2 ^= 0.75) for ^18^F-FDG-PET versus reference volume and y = 1.27x + 56.9 (R^2 ^= 0.80) for caliper versus reference volume.

**Figure 2:**
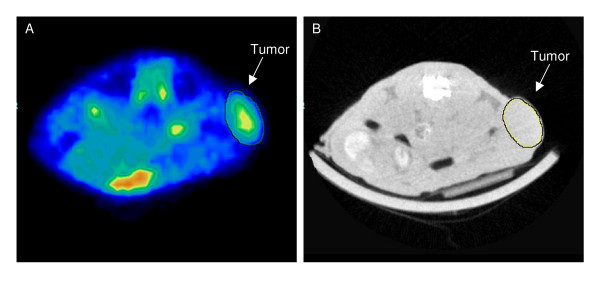
**Transverse section of a representative ^18^F-FDG-PET (A) and microCT (B) image of a mouse with a subcutaneous tumor**. Tumor is indicated by a white arrow and ROIs are drawn separately in the PET and microCT picture.

Tumor volume measured by microCT, PET and caliper all correlated (P < 0.001) with reference volume (figure 1). MicroCT versus reference volume (n = 20) had the best fit of line y = 1.01 ± 0.04x - 6.1 ± 6.3 (R^2 ^= 0.97; p < 0.001). Since 2 tumors were unidentifiable on the PET scan only 18 tumors were available for comparison of ^18^F-FDG-PET with reference volume. The tumors used in the study did not contain necrotic elements. The best line for ^18^F-FDG-PET versus reference volume was y = 1.24 ± 0.18x + 15.4 ± 30.4 (R^2 ^= 0.75; p < 0.001). Caliper versus reference volume (n = 20) had the best fit of line y = 1.27 ± 0.15x + 56.9 ± 24.2 (R^2 ^= 0.80; p < 0.001). An example of ROIs drawn in the microCT and PET pictures is shown in figure 2.

**Figure 3:**
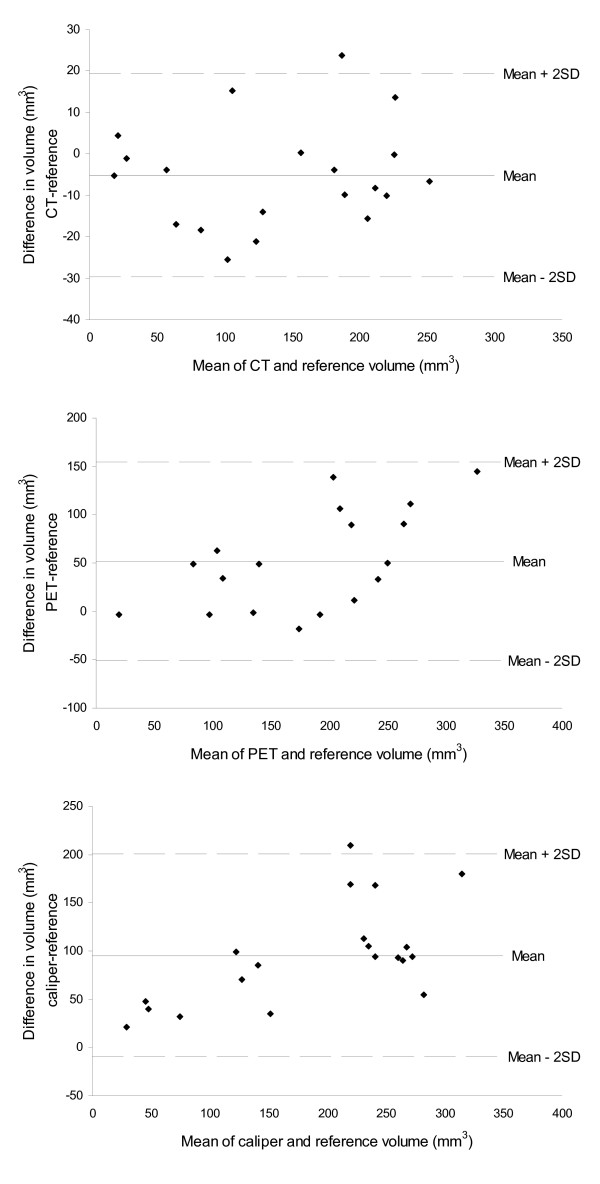
**Bland-Altman plots comparing three methods for measurement of tumor volume of subcutaneous mouse xenografts with the reference volume**. The central line (mean) indicates the bias and the outer lines (± 2SD) indicate the limits of agreement (LoA).

Bland-Altman plots of volume measured by microCT, PET and caliper versus reference tumor volume are shown in figure 3.

The mean difference between microCT and reference tumor volume was -5.1 mm^3 ^(95% CI on difference: -10.6-0.3 mm^3^; LoA: -29.6-19.4 mm^3^). Accordingly, no significant bias of microCT measurements compared with the reference was found. The mean difference between PET and reference tumor volume was 52.1 mm^3 ^(95% CI on difference: 28.0–73.3 mm^3^; LoA: -50.4–154.7 mm^3^) and between caliper and reference tumor volume it was 95.1 mm^3 ^(95% CI on difference: 71.6–118.6 mm^3^; LoA: -10.0–200.3 mm^3^). Accordingly, both PET and caliper had systematic bias when compared to reference volume. The average overestimate of volume using PET was 52 mm^3 ^(35%) and overestimation of volume by using caliper was 95 mm^3 ^(86%).

### Intra- and inter-observer variation

Intra-observer variation expressed as CV was on average 7% (83.4 ± 6.9 mm^3^, n = 10; 93.4 ± 6.1 mm^3^, n = 10; mean ± SD) for the microCT method and 14% (102.4 ± 17.4 mm^3^, n = 10; 167.3 ± 18.0 mm^3^, n = 10; mean ± SD) for the caliper method.

**Figure 4:**
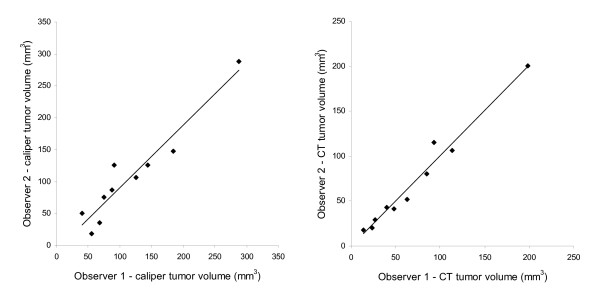
**Correlation of tumor volume determined by two different observers measured by caliper and microCT respectively**. Tumor volumes measures by the two observers were plotted and correlations were evaluated by means of linear fitting and correlation coefficients (R^2^). Best line was y = 0.98x - 7.4 (R^2 ^= 0.91) for caliper and y = 1.01x - 0.65 (R^2 ^= 0.97) for microCT measurements.

**Figure 5:**
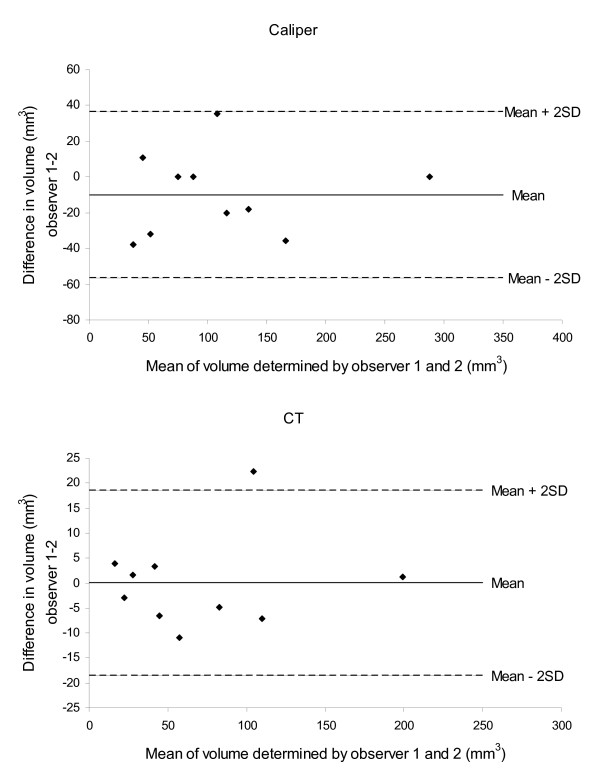
**Bland-Altman plots of the difference between two observers against mean tumor volume**. The central line (mean) indicates the bias and the outer lines (± 2SD) indicate the limits of agreement (LoA).

Inter-observer variation expressed as correlations of tumor volume determined by the two observers are shown in figure 4. Correlation coefficients (R^2^) were 0.97 for microCT and 0.91 for caliper measurements (n = 10). Bland-Altman plots of the difference between two observers against mean volume are shown in figure 5.

The mean difference between observers for caliper measurements was -9.8 mm^3 ^(95% CI on difference: -24.5-4.9 mm^3^; LoA: -56.3-36.7 mm^3^) and for microCT measurements it was 0.0 mm^3 ^(95% CI on difference: -5.9-5.9 mm^3^; LoA: -18.6-18.6 mm^3^). Accordingly, no systematic bias was found between observers.

## Discussion

Most cancer treatment studies assess drug effect by sequential measurements of tumor volume. Currently, the standard method for non-invasive volume measurements of subcutaneous tumors in mice is with external caliper. This is a somewhat subjective method often affected by much error [1] and accordingly there is a need for more accurate volume measurements. Non-invasive imaging modalities such as ultrasonography and MRI have been investigated for their ability to follow tumors in mice during longitudinal studies *in vivo *[10,11]. Ultrasonography and MRI were both shown to be valuable tools for estimating volume of small tumor masses. Ultrasonography has the advantage of having a relative low cost of equipment and both MRI and ultrasonography have the advantage that they do not impose any radiation dose that may interfere with tumor growth. Further, it has been shown that CT as part of a clinical PET/CT scanner can determine volume more precisely than traditional caliper measurements of large subcutaneous tumors in rats [12]. Preclinical imaging of small animals with dedicated animal microCT and microPET scanners has in recent years become available and could be even better alternatives for tumor volume determination. Accordingly, we evaluated the capability of these modalities for volume determination of subcutaneous tumors in mice.

We found that the microCT method was altogether more accurate than both PET and caliper methods for determination of tumor volume. For the microCT method there was no systematic bias, whereas both the PET and caliper method systematically overestimated tumor size. The bias of the caliper measurements was smaller for small tumors compared to greater tumors also relatively seen. Consequently, volume changes measured with caliper in small and large tumors are not comparable and effects of anti-cancer drugs can easily be missed as tumors will tend towards being determined with a greater bias as they grow larger. The bias in PET measurements was not dependent on tumor size.

Intra-observer variation on the microCT measurements was substantially lower compared to variation on the caliper measurements. In consequence, microCT measurements will allow for detection of smaller changes and earlier recognition of efficacy in subcutaneous xenografts during experimental cancer treatment studies than standard external caliper. Further, it will allow for reduction in the number of animals necessary to show a given effect in cancer treatment studies *e.g. *when testing new anticancer drugs.

Previous, analysis of intra-observer variation of caliper measurements have been carried out. CV was 12% for a small (320 mm^3^) and 6% for a larger (1450 mm^3^) tumor [1]. Using even smaller tumors (70–90 mm^3^) we found CV to be 14% for the caliper method and accordingly overlooking effects during longitudinal treatment studies can be marked with this method. In contrast, in the present study, where small tumors were used for the intra-observer variation, we fund a CV for microCT measurements as low as 7%. MicroCT hence allows detection of small changes in much smaller tumors than the traditional caliper.

Tumor volume was determined with a rather high reproducibility between observers by both the microCT and caliper methods. Variation between observers for microCT measurements in this study was comparable to a study of much greater tumors in rats [12], however the current study has showed that the low inter-observer variation of the microCT method is also valid for small tumors.

Volume determination by the caliper method was inaccurate with a significant bias that increased with tumor size. Very likely, this inaccuracy is partly due to the assumption that all tumors have shape like a modified ellipsoid, which may be less true for large tumors. With the microCT method, bias that arises from assumption of this specific geometry is removed. In consequence, tumor volume is accurately determined irrespective of tumor size and form.

Tumor volume measured by microPET did not correlate well with true tumor volume. In order to determine tumor size by ^18^F-FDG-PET, all parts of the tumor must take up ^18^F-FDG. Visual inspection of PET images in this study (figure 2) showed a heterogeneous ^18^F-FDG uptake in the tumors, which made it difficult accurately to identify tumor boundary. The resolution of the microPET images was not as high as of the microCT images which also contributed to the much lower accuracy of the PET volume data. Therefore, it was not unexpected that ^18^F-FDG-PET was unsuitable for determination of tumor volume and consequently ^18^F-FDG-PET is rarely used for volume measurements. As the current study showed that the microCT method accurately and precisely identified tumor volumes, identification of tumors based on the anatomically CT image and subsequently fusion of PET and CT images will allow much more precise determination of tracer uptake. Accordingly, a combination of microCT with microPET will allow a sensitive and accurate quantification of tumor burden in mice and be valuable for the evaluation of novel cancer treatments.

## Conclusion

In summary, the present study demonstrated that microCT was more accurate than both external caliper measurements and ^18^F-FDG-microPET for *in vivo *volumetric measurements of subcutaneous tumors in mice.^18^F-FDG-microPET was considered unsuitable for determination of tumor size. External caliper were inaccurate and encumbered with a significant and size dependent bias. External caliper are, despite this inaccuracy, currently the standard method for determination of tumor volume due to the low cost and high throughput of the simple method. In contrast, we found that microCT was accurate, without systematic bias and more reproducible than caliper measurements. Consequently, microCT is a promising method that should be used when studies of small changes in experimental cancer treatment studies of subcutaneous tumors in mice is needed.

## Abbreviations

CI: Confidence interval; CT: Computed tomography; CV: Coefficient of variation; ^18^F-FDG: ^18^F-fluorodeoxyglucose; LoA: Limits of agreement; PET: Positron emission tomography; ROI: Region of interest; SUV: Standard uptake value.

## Competing interests

The authors declare that they have no competing interests.

## Authors' contributions

MMJ: conception and design of the study, animal studies, image and data analysis, draft of manuscript. JTJ and TB: animal studies and image analysis. AK: conception and design of the study, draft of manuscript. All authors read and approved the final manuscript.

## Pre-publication history

The pre-publication history for this paper can be accessed here:


